# The Bidirectional Relationship Between Serum Uric Acid and Hypertension

**DOI:** 10.1155/ijhy/3022250

**Published:** 2026-11-04

**Authors:** Xiang Shu, Kaiwen Deng, Min Zhang, Bo Li, Dongqing Gu, Wantai Dang, Weizhong Chen, Ziqian Zeng

**Affiliations:** ^1^ Department of Epidemiology and Health Statistics Chengdu Medical College Chengdu China cmc.edu.cn; ^2^ Women and Children’s Hospital of Chongqing Medical University Chongqing China cqmu.edu.cn; ^3^ Clinical Pharmacy Department The 3RD Affiliated Hospital of Chengdu Medical College Chengdu Pidu District People’s Hospital Chengdu Sichuan China; ^4^ Department of Rheumatology and Immunology The First Affiliated Hospital of Chengdu University of Chinese Medicine Chengdu China

**Keywords:** body mass index, hypertension, mediation mendelian randomization, serum uric acid

## Abstract

**Background:**

Hypertension (HTN) may influence other chronic diseases, and a bidirectional relationship between HTN and serum uric acid (SUA) has been suggested, but evidence is insufficient. Body mass index (BMI) is associated with both HTN and SUA levels and may mediate their causal relationship. This study investigates the bidirectional causal relationship between HTN and SUA levels and the mediating effects of BMI.

**Methods:**

The two‐sample bidirectional and mediation Mendelian randomization (MR) studies were conducted. In the first part, we conducted the univariable and multivariable bidirectional MR analyses. In the second part, BMI was included in the MR analyses as a mediator. Summary data on HTN, SUA, and BMI were obtained from the FinnGen, GWAS Catalog, and IEU OpenGWAS database.

**Results:**

The sample sizes of the European population included in analyses were 478,149 individuals for HTN, 343,836 for SUA, and 99,998 for BMI. In the European population, genetically predicted higher SUA was associated with increased HTN risk (OR = 1.215; 95% CI: 1.119 ∼ 1.320). HTN had an effect on SUA (OR = 1.018; 95% CI: 1.003 ∼ 1.033), in which the indirect effect via BMI showed an estimate of 0.006 (95% CI, 0.002 ∼ 0.010) with the mediated proportion of 27.3% (95% CI, 5.6% ∼ 48.9%).

**Conclusion:**

A bidirectional relationship between SUA and HTN was identified in the European population, with BMI partially mediating the effect of HTN on SUA.

## 1. Introduction

Hypertension (HTN) is defined as the diastolic and systolic blood pressure in the arteries being continually raised or taking hypotensive drugs for maintaining the normal blood pressure [1]. The data from the latest research showed that no less than 1.28 billion people aged 30 ∼ 79 years worldwide have already been diagnosed with HTN, which poses a significant challenge to global public health [2]. In addition, HTN is widely acknowledged as an important risk factor of cardiovascular diseases, which could cause high mortality and morbidity [3, 4].

Serum uric acid (SUA), a final product of purine catabolism in the human body, was recognized to be related to human health. In recent years, along with the change of people’s dietary patterns, the prevalence of hyperuricemia (HUA) has gradually increased [5, 6]. A large number of studies reported that HUA is associated with HTN [7–9]. Data from the Uric Acid Right for Heart Health (URRAH) project provide further support for the clinical relevance of SUA, demonstrating that elevated SUA is independently associated with all‐cause mortality, cardiovascular mortality, and major adverse cardiovascular events [10]. In addition, the possible mechanism of raising blood pressure by HUA may include stimulating the proliferation of vascular smooth muscle cells, causing vascular inflammation, damaging endothelial cells, etc. [7, 8]. However, due to the confounding factors, most of the previous association studies presented inconsistent results [11].

Moreover, few researchers reported that the high blood pressure might also be the influencing factor of SUA levels, but the conclusions were also not consistent in different populations. For example, a cohort study conducted in Japan reported that HTN was one of the risk factors for HUA among men, and the difference was not found in the female population [12]. Similarly, two other cohort studies conducted among middle‐aged men in Japan and South Korea reported that high blood pressure had a higher risk of HUA [13, 14].

Besides the direct association between SUA and HTN, the indirect relationship might also exist. Body mass index (BMI) was recognized as an important factor related to both HTN and SUA levels in many studies [12, 15, 16]. In addition, BMI could also be influenced by SUA levels [17]. Meanwhile, BMI might have a mediated effect and should not be treated as a confounding factor, which was popular in previous studies [18]. Presently, there is still lack of evidence to clarify whether BMI plays a role in the pathway from SUA to HTN, given that BMI is a shared upstream risk factor for both conditions. In addition, it remains unclear whether HTN‐induced changes in metabolic status exert part of their influence on SUA levels through alterations in BMI‐related pathways. There is still insufficient evidence to clarify this mediating effect in the context of the bidirectional relationship between SUA and HTN.

Based on the above discussion, this study hypothesizes that SUA and HTN have a bidirectional causal relationship and that BMI may act as a mediating variable in the pathway. Mendelian randomization (MR) study is used to test the hypothesis in this study, which is a tool utilized frequently for causal inference using single nucleotide polymorphisms (SNPs) as instrumental variables (IVs) [19, 20]. The findings could provide valuable insights for the prevention and treatment of both HUA and HTN.

## 2. Methods

### 2.1. Study Design

We used the two‐sample MR to evaluate the bidirectional causal relationship between SUA and HTN, and multivariate MR, along with the two‐step MR, was employed to assess the mediating effect of BMI using the delta method. The MR was conducted based on three basic assumptions: (1) the genetic variants must be highly associated with exposure; (2) genetic variants cannot be corrected with any potential confounding factors; and (3) genetic variants influence outcome solely through the exposure.

### 2.2. Data Sources

Genome‐wide association study (GWAS) data was used to conduct the MR analyses. To prevent bias caused by sample overlap, the study obtained exposure and outcome data from multiple databases. As this study was based on publicly available GWAS summary statistics, missing data at the individual level were already handled in the original GWAS analyses. For the European population, the summary statistics for BMI (*N* = 99,998) were obtained from the IEU Open GWAS project (https://gwas.mrcieu.ac.uk), HTN (*N* = 478,149, including 132,515 cases and 345,634 controls) were sourced from the FinnGen database (https://www.finngen.fi/en), and SUA level (*N* = 343,836) was downloaded from the GWAS Catalog (https://www.ebi.ac.uk/gwas/). All data above are from the public database, and the studies have obtained ethical approval and do not require additional separate approval (Table 1, Supporting Table 1).

**TABLE 1:** Data sources for the European population included in the MR analyze.

Variable	HTN	SUA	BMI
Database	FinnGen	GWAS Catalog	IEU OpenGWAS
Population	European	European	European
Sample size	478,149	343,836	99,998
No. of cases	132,515	343,836	99,998
No. of controls	345,634	—	—
SNPs	21,326,806	19,041,286	7,191,606

### 2.3. IVs Selection

Genetic variants were selected as IVs according to the following criteria: (1) significant association with the exposure at a *p* < 5 × 10^−8^, (2) a linkage disequilibrium *r* ^2^ < 0.001, or a genetic distance ≤ 10000 kb, (3) minimum allele frequency > 0.01, (4) exclusion of SNPs with palindromic intermediate allele frequencies, and (5) independence of the SNPs from each other. After screening according to the criteria, this study finally included 113 SNPs for European ancestry highly related to SUA from GWAS data as IVs, and 27 SNPs related to BMI were included as IVs to study the relationship between BMI and HTN. 108 SNPs highly related to HTN were included as IVs to study the relationship between HTN and SUA. 28 SNPs related to BMI were included as IVs to conduct research on BMI and SUA. In this study, F‐statistic exceeded 10 for all SNPs, indicating the absence of weak IV bias.

### 2.4. Statistical Analysis

We used the Wald ratio to estimate the causal impact of each IV. An inverse‐variance weighted (IVW)‐based multiplicative random‐effects model was employed [21]. First, the five techniques (MR‐Egger, weighted median, simple mode, weighted mode and IVW) were employed to assess the association between genetically predicted SUA‐associated features and the risk of HTN [22]. The results were visualized using forest and scatter plots. In addition, in the scatterplot, estimated regression lines from the multiple SNP analysis are overlaid to compare the impact of single SNPs on exposure and outcomes. Second, a series of sensitivity analyses were conducted. Multiple methods including the weighted median method, MR‐Egger, simple mode, and weighted mode to provide estimates under a wider range of conditions. Moreover, horizontal multiplicity was evaluated through MR‐Egger intercept data [23]. The challenges posed by complex behavioral exposures make it difficult for commonly used pleiotropy robust methods to yield estimates with the correct direction of effect, highlighting the struggle in detecting systematic pleiotropy. These estimators often assume that each SNP has a unique pleiotropic effect. Our study exercised caution in interpreting results within such settings. Considering the systematic pleiotropy, a leave‐one‐out analysis using the IVW MR method was used to investigate the robustness of findings. The funnel plots were used to assess heterogeneity. More details could be found in Supporting Figures 1–4.

The robustness of MR results was tested by sensitivity analyses which were conducted by removing 1 variant at a time and recalculating the overall MR estimate.

Two‐step MR and sobel tests (https://quantpsy.org/sobel/sobel.htm) were used for Mediation analysis. The IVW method was applied to evaluate the causal effects.

The effect estimates were expressed as odds ratios (ORs) and 95% confidence intervals (CIs) per standard deviation (SD) increase in the exposure.

All these analyses were performed using R (Version 4.3.1) with the packages ‘MR‐PRESSO’, ‘Two sample MR’, and ‘MVMR’, and ‘RMediation’. Statistical significance was defined as *p* < 0.05.

## 3. Results

### 3.1. The Bidirectional Association Between SUA and HTN Using Univariable MR

The results from univariable MR indicated that SUA was associated with HTN in the European population(OR = 1.230; 95% CI: 1.157 ∼ 1.308). In addition, the association between HTN and SUA was weak but statistically significant (OR = 1.022; 95% CI: 1.012 ∼ 1.032). The results are shown in Figure 1.

**FIGURE 1:**
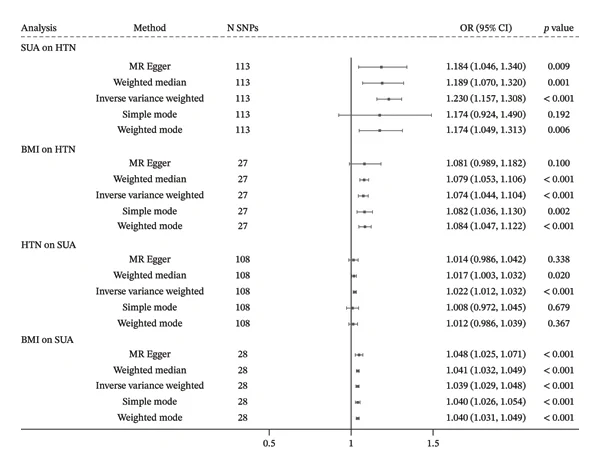
The results of univariable MR analyses in European population.

### 3.2. The Bidirectional Association Between SUA and HTN Using Multivariable MR

The results of multivariate MR presented that in the European population, after adjusting for BMI, the effect of SUA on HTN remained statistically significant (OR = 1.215; 95% CI: 1.119 ∼ 1.320), and the effect of HTN on SUA was also observed (OR = 1.018; 95% CI: 1.003 ∼ 1.033) (Figure 2).

**FIGURE 2:**
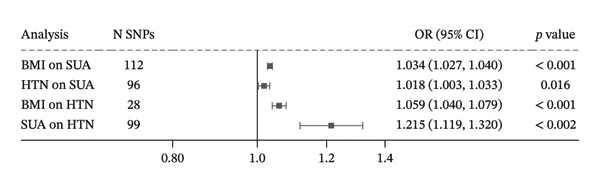
The results of multivariable MR analyses in European.

### 3.3. The Mediating Effect of BMI

The role of BMI in the association between SUA and HTN was further explored in this study. Mediation MR analyses indicated that in the European population, HTN had an indirect effect on SUA through BMI, with a mediating effect of 0.006 (95% CI: 0.002 ∼ 0.010) and a mediated proportion of 27.3% (95% CI: 5.6% ∼ 48.9%). However, no statistically significant mediation effect was observed for the SUA‐BMI‐HTN pathway (Table 2).

**TABLE 2:** The results from mediation MR in European.

Path	Total effect β (95% CI)	Direct effect A β (95% CI)	Direct effect B β (95% CI)	Mediation effect β (95% CI)	*p*	Mediated proportion (%)
SUA‐BMI‐HTN	0.207 (0.145, 0.269)	0.023 (−0.095, 0.140)	0.071 (0.043, 0.099)	0.002 (−0.007, 0.010)	0.708	1.0 (−3.2, 5.1)
HTN‐BMI‐SUA	0.022 (0.012, 0.031)	0.145 (0.051, 0.239)	0.038 (0.029, 0.047)	0.006 (0.002, 0.010)	< 0.001	27.3 (5.6, 48.9)

*Note:* The total effect was estimated using univariable Mendelian randomization. Direct effect A represents the causal effect of the exposure on BMI, whereas Direct effect B represents the causal effect of BMI on the outcome. The indirect (mediation) effect was calculated using the product‐of‐coefficients method by multiplying Direct effect A and Direct effect B. The mediated proportion was estimated as the ratio of the indirect effect to the total effect using the delta method.

### 3.4. Sensitivity Analyses

To assess the robustness of the MR results, sensitivity analyses were performed using the selected SNPs. In the European population, Cochran’s Q test showed no evidence of statistically significant heterogeneity across any of the four causal pathways (*p* > 0.05; Table 3). Furthermore, the MR‐PRESSO global test and MR‐Egger intercept test revealed no evidence of statistically significant horizontal pleiotropy (*p* > 0.05; Table 3). Leave‐one‐out analyses further supported the stability of the MR estimates (Supporting Figures 1 to 4).

**TABLE 3:** The results of sensitivity analyses in European.

Exposure	Outcome	MR‐PRESSO test	MR‐Egger test	Cochran Q test
BMI	SUA	0.316	0.773	0.209
HTN	SUA	0.074	0.956	0.057
BMI	HTN	0.873	0.770	0.714
SUA	HTN	0.287	0.411	0.261

## 4. Discussion

This study provides novel and comprehensive evidence to the relationship between SUA and HTN. Previous studies have indicated that the bidirectional relationship between SUA and HTN [7–9, 11–14] might be possible; however, these results still lack concrete evidence. Although several studies including MR analysis had explored the association between SUA and HTN, only few studies paid attention to the reverse effects of HTN on SUA. A recent MR study by Xu et al. similarly confirmed a unidirectional causal effect of SUA on HTN risk in European populations [24]. The present study extends those findings in two important ways: first, by demonstrating a bidirectional relationship in which HTN also causally elevates SUA; and second, by investigating the potential role of BMI as an intermediate variable specifically in the HTN‐to‐SUA pathway. It should be acknowledged that BMI is an upstream shared risk factor for both SUA and HTN; however, given the physiological evidence that HTN promotes adiposity through neuroendocrine mechanisms and metabolic dysregulation, we explored whether BMI may also partially transmit the effect of HTN on SUA levels. In addition, the BMI, as an important confounding factor, was always treated as a confounding factor by adjusting in regression models, but the potential for BMI to operate as an intermediate variable on the specific HTN‐to‐SUA pathway had not been formally examined. In this study, we firstly performed the univariable and multivariable MR to confirm the bidirectional association between SUA and HTN and then conducted the mediation MR to examine whether BMI partially transmits the effect of HTN on SUA. The clinical significance of these findings lies in their implications for disease management: if elevated SUA not only results from, but also contributes to, HTN through a bidirectional loop partly mediated by adiposity‐related pathways, this supports integrated strategies targeting both uric acid and body weight in the prevention and treatment of HTN and its metabolic complications.

Previous observational studies have explored the association between SUA and HTN. The results from the Chinese population with a large sample size indicated that higher SUA could increase the risk of HTN [25, 26], and similar findings were reported by studies targeting other populations [27, 28]. In addition, the dose‐response relationship between them was also found in a systematic review that included 17 prospective cohorts with 32,000 participants [29]. There were also some findings studying the relationship between HTN and SUA. The study from India reported the HTN as a determinant of HUA [30]; however, the association was not found in Ethiopian populations [31]. These studies provided inconsistent and confusing results, probably because of small sample size, selection bias, information bias, and confounding bias.

To reduce the confounding bias, the MR studies, considering the random segregation of genetic alleles when parents transmit their DNA to offspring, were applied to provide a higher level evidence [32]. Currently, some researchers have conducted MR studies to test the causal association between SUA and HTN. However, some of these results were inconsistent with these observational studies [7, 33]. This study found a bidirectional relationship between HTN and SUA in the European population. BMI is one of the important risk factors for several chronic diseases, including SUA and HTN [34, 35]. Based on this evidence, we conducted a mediation MR analysis to explore the potential role of BMI as an intermediate variable in the HTN‐to‐SUA pathway. We recognize that BMI is a common upstream risk factor for both SUA and HTN; however, the pathophysiological literature also supports a downstream role for HTN in promoting adiposity: chronic HTN activates the renin‐angiotensin‐aldosterone system and sympathetic nervous system, which may in turn promote visceral fat accumulation and metabolic changes that elevate SUA. The results indicated that both SUA and BMI could influence HTN directly, and in the European population, HTN had an indirect effect on SUA through BMI, consistent with this mechanistic rationale. It should also be acknowledged that BMI is a relatively crude measure of overall adiposity and does not adequately capture body fat distribution or visceral adiposity. A previous study reported significant associations between SUA and alternative adiposity indices, with LAP showing a particularly strong association with HUA [36]. Accordingly, future studies incorporating more specific indices of visceral and metabolic adiposity may help clarify the adiposity‐related pathways underlying the relationship between HTN and SUA.

Our study had several strengths. First, MR analysis leverages the principle of random segregation of genetic alleles at conception as a form of natural randomization, thereby providing evidence for potential causal associations between risk factors and diseases while reducing the influence of confounding and reverse causality that often affect conventional observational studies. It is important to note that MR estimates represent the effect of genetically predicted (lifelong) exposure levels on outcomes and should be interpreted as reflecting causal relationships at the genetic instrument level, rather than direct intervention effects. Using existing large‐scale GWAS data, this study investigated the causal relationship between SUA and HTN. In addition, no evidence of heterogeneity or pleiotropy was detected in the analyses, supporting the robustness of our findings. Second, most previous studies mainly focused on the association between SUA and HTN, whereas the relationship between HTN and SUA remained unclear. This study further clarified the causal relationship between HTN and SUA in the European population, extending the recent unidirectional MR evidence by Xu et al. (2025) to confirm bidirectionality [24]. Third, this study also provided evidence that BMI may partially transmit the effect of HTN on SUA in the European population, a finding that, if confirmed, would have implications for integrated clinical management of HTN and HUA.

Several limitations should be acknowledged. First, data quality control standards may not have been unified across source populations, potentially introducing heterogeneity in the GWAS summary statistics. Second, individual‐level data were unavailable, precluding adjustment for covariates such as sex, pharmacological treatment, and lifestyle factors. Third, although diuretics, particularly thiazides, can substantially elevate SUA by inhibiting renal tubular urate excretion [37], they do not act as classical confounders within the MR framework, since genetic variants are fixed at conception prior to any drug exposure. They may, however, function as effect modifiers. Because individual‐level medication data were unavailable, stratified analyses by diuretic use could not be performed and should be addressed in future studies. Fourth, BMI is a shared upstream risk factor for both HTN and SUA, which complicates causal interpretation of the mediation estimate; this finding should be regarded as exploratory and requires independent replication. Fifth, other biological and metabolic factors potentially contributing to the bidirectional relationship were not systematically evaluated and warrant further investigation.

## 5. Conclusions

In summary, this study identified a bidirectional causal relationship between SUA and HTN in the European population and further suggested that BMI may partially mediate the effect of HTN on SUA.

**Nomenclature**
BMI: Body mass index
CI: Confidence interval
GWAS: Genome‐wide association study
HR: Hazard ratio
HTN: Hypertension
HUA: Hyperuricemia
IV: Instrumental variable
IVW: Inverse‐variance weighted
MR: Mendelian randomization
MR‐PRESSO: Mendelian Randomization Pleiotropy RESidual Sum and Outlier test
MVMR: Multivariable Mendelian randomization
OR: Odds ratio
SNP: Single nucleotide polymorphism
SUA: Serum uric acid

## Author Contributions

Xiang Shu, Kaiwen Deng, Min Zhang, and Bo Li contributed equally to this work. Xiang Shu and Kaiwen Deng were responsible for software implementation, formal analysis, and manuscript drafting. Min Zhang contributed to software implementation, visualization, and manuscript drafting. Bo Li contributed to software implementation, result validation, and manuscript revision. Dongqing Gu was responsible for data curation and contributed to manuscript revision. Wantai Dang participated in manuscript review and editing. Weizhong Chen was responsible for methodology and contributed to manuscript revision. Ziqian Zeng was responsible for study conceptualization, supervision, funding acquisition, and manuscript revision.

## Funding

This work was supported by the Open Project of the Sichuan Collaborative Innovation Center for Aging and Elderly Health (grant number 23LHPDZYB23).

## Disclosure

All authors reviewed and approved the final manuscript.

## Ethics Statement

This study is a Mendelian randomization analysis using publicly available GWAS summary‐level data. All original studies had obtained ethical approval and informed consent. As this study involved only secondary analyses of anonymized summary data, no additional ethical approval was required.

## Conflicts of Interest

The authors declare no conflicts of interest.

## Supporting Information

Additional supporting information can be found online in the Supporting Information section.

## Supporting information


**Supporting Information** Supporting Table 1: GWAS data sources used in this study (key elements of study design and data sources). Supporting Figure 1 MR analysis of the causal effect of HTN on SUA in the European population. Supporting Figure 2: MR analysis of the causal effect of BMI on SUA in the European population. Supporting Figure 3: MR analysis of the causal effect of SUA on HTN in the European population. Supporting Figure 4: MR analysis of the causal effect of BMI on HTN in the European population.

## Data Availability

The data that support the findings of this study are available in the IEU OpenGWAS project (https://gwas.mrcieu.ac.uk), the FinnGen database (https://www.finngen.fi/en), and the GWAS Catalog (https://www.ebi.ac.uk/gwas/). Specifically, for the European population, BMI data were obtained from the IEU OpenGWAS project (ID: ieu‐b‐4816), hypertension data were sourced from the latest FinnGen hypertension dataset (Endpoint ID: I9), and serum uric acid data were retrieved from the GWAS Catalog (ID: GCST90018977).
