# Supplementary material for: The Bidirectional Relationship Between Serum Uric Acid and Hypertension
Source: Int J Hypertens. 2026 Nov 4;2026:3022250. doi: 10.1155/ijhy/3022250 (PMC13736168; doi:10.1155/ijhy/3022250)
Supplement: Supplementary file 1 [file IJHY-2026-3022250-s001.docx]

**Supplementary Table 1. GWAS data sources used in this study (key elements of study design and data sources)**

| Population | Trait | a) Study background | b) Participants | c) Genetic variant measurement and selection | d) Trait measurement and diagnostic criteria | e) Ethics |
| --- | --- | --- | --- | --- | --- | --- |
| European | BMI | GWAS of body mass index (BMI), European ancestry, Within Family GWAS consortium, 2022, build HG19/GRCh37 | *N*=99,998, males and females; publicly available summary statistics; no additional power calculation performed | 7,191,606 SNPs; SNPs for MR selected with *P*<5×10⁻⁸, LD *r²*<0.001 (10,000 kb), MAF>0.01; palindromic SNPs excluded | BMI calculated as weight/height² (kg/m²), analyzed as a continuous trait; no clinical obesity diagnosis applied | Original GWAS obtained ethics approval and informed consent; only de-identified summary statistics were used in this study |
| European | Hypertension (HTN) | Nationwide Finnish biobank-based GWAS, circulatory system diseases (ICD I10–I15), R9 release (2023), build GRCh38 | *N*=478,149 (132,515 cases; 345,634 controls), European ancestry; cases ascertained from Finnish health registries; no additional power calculation performed | Genome-wide genotyping and imputation; QC performed by FinnGen; MR SNPs filtered with *P*<5×10⁻⁸, LD *r²*<0.001, MAF>0.01; palindromic SNPs excluded | Hypertension diagnosis based on ICD codes (I10–I15) from Finnish hospital discharge, primary care, medication reimbursement, and death registries | FinnGen approved by the Ethics Committee of the Hospital District of Helsinki and Uusimaa (HUS); participants provided biobank consent; only de-identified summary statistics used |
| European | Serum uric acid (SUA) | GWAS of SUA levels, European ancestry, published 2021 (PMID: 34594039), build HG19/GRCh37 | *N*=343,836, males and females, European ancestry; publicly available summary statistics; no additional power calculation performed | 19,041,286 SNPs; MR SNPs selected with *P*<5×10⁻⁸, LD *r²*<0.001 (10,000 kb), MAF>0.01; palindromic SNPs excluded | SUA levels measured as continuous serum biochemical trait; no clinical hyperuricemia diagnosis used | Original GWAS obtained ethics approval and participant consent; only de-identified summary statistics used in this study |

**Supplementary Figure 1. MR analysis of the causal effect of HTN on SUA in the European population.** Panel A shows the scatter plot of SNP-specific causal estimates. Panel B presents the forest plot of individual SNP effects. Panel C illustrates the leave-one-out sensitivity analysis, demonstrating the robustness of the MR estimates. Panel D shows the funnel plot used to assess heterogeneity and potential horizontal pleiotropy. Abbreviations: SUA, serum uric acid; HTN, hypertension; MR, Mendelian randomization; SNP, single-nucleotide polymorphism.

**Supplementary Figure 2. MR analysis of the causal effect of BMI on SUA in the European population.** Panel A shows the scatter plot of SNP-specific causal estimates. Panel B presents the forest plot of individual SNP effects. Panel C illustrates the leave-one-out sensitivity analysis, demonstrating the robustness of the MR estimates. Panel D shows the funnel plot used to assess heterogeneity and potential horizontal pleiotropy. Abbreviations: SUA, serum uric acid; HTN, hypertension; MR, Mendelian randomization; SNP, single-nucleotide polymorphism.

**Supplementary Figure 3. MR analysis of the causal effect of SUA on HTN in the European population.** Panel A shows the scatter plot of SNP-specific causal estimates. Panel B presents the forest plot of individual SNP effects. Panel C illustrates the leave-one-out sensitivity analysis, demonstrating the robustness of the MR estimates. Panel D shows the funnel plot used to assess heterogeneity and potential horizontal pleiotropy. Abbreviations: SUA, serum uric acid; HTN, hypertension; MR, Mendelian randomization; SNP, single-nucleotide polymorphism.

**Supplementary Figure 4. MR analysis of the causal effect of BMI on HTN in the European population.** Panel A shows the scatter plot of SNP-specific causal estimates. Panel B presents the forest plot of individual SNP effects. Panel C illustrates the leave-one-out sensitivity analysis, demonstrating the robustness of the MR estimates. Panel D shows the funnel plot used to assess heterogeneity and potential horizontal pleiotropy. Abbreviations: SUA, serum uric acid; HTN, hypertension; MR, Mendelian randomization; SNP, single-nucleotide polymorphism.
